# A first-principle mechanism for particulate aggregation and self-assembly in stratified fluids

**DOI:** 10.1038/s41467-019-13643-y

**Published:** 2019-12-20

**Authors:** Roberto Camassa, Daniel M. Harris, Robert Hunt, Zeliha Kilic, Richard M. McLaughlin

**Affiliations:** 1 Department of Mathematics, University of North Carolina, Chapel Hill, Chapel Hill, NC 27599 USA; 2 School of Engineering, Brown University, Providence, RI 02912 USA; 3 Department of Physics and Center for Biological Physics, Arizona State University, Tempe, AZ 85287 US

**Keywords:** Applied mathematics, Fluid dynamics

## Abstract

An extremely broad and important class of phenomena in nature involves the settling and aggregation of matter under gravitation in fluid systems. Here, we observe and model mathematically an unexpected fundamental mechanism by which particles suspended within stratification may self-assemble and form large aggregates without adhesion. This phenomenon arises through a complex interplay involving solute diffusion, impermeable boundaries, and aggregate geometry, which produces toroidal flows. We show that these flows yield attractive horizontal forces between particles at the same heights. We observe that many particles demonstrate a collective motion revealing a system which appears to solve jigsaw-like puzzles on its way to organizing into a large-scale disc-like shape, with the effective force increasing as the collective disc radius grows. Control experiments isolate the individual dynamics, which are quantitatively predicted by simulations. Numerical force calculations with two spheres are used to build many-body simulations which capture observed features of self-assembly.

## Abstract (web summary)

Aggregation of matter, common in stratified fluid systems, is essential to the carbon cycle and ocean ecology. Although the current understanding of aggregation involves only collision and adhesion, here Camassa et al. reveal a self-assembly phenomenon arising solely from diffusion-induced flows.

## Introduction

Particle sedimentation and aggregation in stratified fluids is observed throughout natural systems. Some examples include: sedimenting “marine snow” particles in lakes and oceans (central to carbon sequestration)^1^, dense microplastics in the oceans (which impact ocean ecology and the food chain^2^), and even “iron snow” on Mercury^3^ (conjectured as its magnetic field source). These fluid systems all have stable density stratification, which is known to trap particulates^1,4–7^ through upper lightweight fluid coating the sinking particles, thus providing transient buoyancy. Numerous mechanisms have been described by which particles may aggregate in such environments. The current understanding of aggregation of such trapped matter involves collisions (owing to Brownian motion, shear, and differential settling) and adhesion^8,9^.

In this work, we identify and isolate an unexplored mechanism, which induces particle attraction and self-assembly in stratified fluids in the absence of adhesion (short range binding effects) arising as a first-principle fluid dynamics phenomenon. Specifically, we present first an experiment, which documents the self-assembly with hundreds of particles. We next present a series of control experiments and calculations to explain the underlying mechanism responsible for the attraction. This involves using Lagrangian tracer particle dynamics to observe flows created by single bodies of different aspect ratios. In turn, we use particle imaging velocimetry (PIV) to observe the flow structure in a plane induced by a single spheroid. We compare these experimental observations quantitatively with simulations performed within the COMSOL software environment. With validated simulations, we are able to further explore the flow structure’s dependence on physical parameters (aspect ratio, size, density gradient, etc). To explore many-body effects, we first simulate the flow induced by two fixed, same size spheres and evaluate numerically the induced force at an array of separation distances. This resulting force law is used to develop a modified Stokesian dynamics simulation (capable of simulating hundreds of spheres), which is in turn validated with the experiments involving two same size, moving spheres. Modified Stokesian dynamics results are then presented for hundreds of spheres exhibiting self-assembly features which strongly resemble those observed in our many-body experiment.

## Results

### Experimental observations

**Fig. 1:**
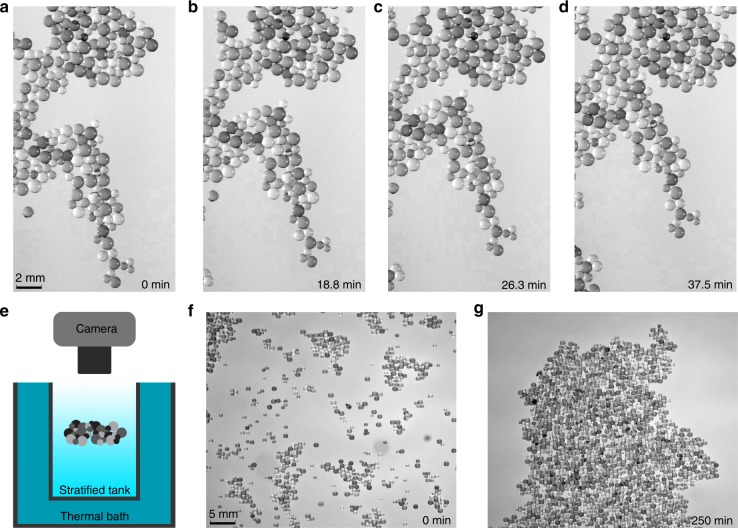
Experimental snapshots and schematic. **a**–**d** Time series of self assembly of collection of neutrally buoyant spheres suspended within a sharply salt-stratified fluid viewed from above. Spheres radii and densities are 0.025–0.05 cm and $1.05$ g cc^−1^, top fluid is fresh water ($0.997$ g cc^−1^), bottom is NaCl water solution of density ($1.1$ g cc^−1^). **e** Schematic showing experimental setup. **f** Initial cluster (different trial). **g** Final cluster.

The self-assembly phenomenon is clearly observed in our experiments (Fig. 1a–d, f, g) with a collection of neutrally buoyant, small spheres suspended at the same height between layers of sharply salt-stratified water in a rectangular plexiglass container. The spheres are photographed from above (see schematic in Fig. 1e) at regular time intervals. The spheres, initially isolated, feel a mutually attractive force, which forms local clusters. In turn, these clusters attract each other while orienting themselves to seemingly try to fill a puzzle-like pattern, ultimately resulting in a large disc-like shape. See Supplementary Movie 1 for a dynamic view of this self-assembling cluster.

**Fig. 2:**
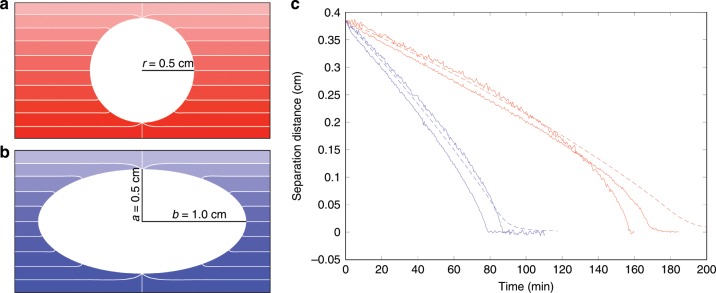
Experiments and computations with large sphere/spheroid. **a**, **b** Side view geometries, and numerically computed density isolines, $Pe=41$, sphere radius, $0.5$ cm, spheroid width, 2 cm, density increments ~$0.001$ g cc^−1^, with density gradient $\sigma =0.007$ g cm^−4^. **c** Experimental and computational evolution of separation distance as a function of time between a large sphere and a small passive sphere and between a large spheroid and a small passive sphere. All experimental trajectories are plotted with the same initial separation. Also shown are the companion trackings produced by our computational simulation for the two geometries (dashed lines). The diffusion-induced flows generated by either large body advect and attract the small passive particle until contact. Note that the velocities in the case of the oblate spheroid are larger than the case of the sphere.

To probe more directly the nature of the interaction between spheres, we next examine cases involving single large bodies interacting with single small bodies in linear stratification (as opposed to the sharp, two-layer stratification in Fig. 1), which can be assumed to be advected by the fluid flow induced by each individual large body (the mechanism for these flow is theoretically explained below). We note that linear concentration gradients are known to be exact solutions to the diffusion equation in free space, which thereby reduces any effect of the background concentration field changing during the duration of these control experiments. All bodies have the same approximate densities ($1.05$ g cc^−1^) and float at similar heights within the salt-stratified layer (density gradient ~ $0.007$ g cm^−4^). The left panel of Fig. 2 shows the side view of the two different large bodies considered. The sphere has radius $0.5$ cm. The oblate spheroid has the same vertical radius, while its horizontal radius is $1.0$  cm. We monitor the separation distance as a function of time between the large body and the small body. In the right panel of Fig. 2, we present these trackings for the case of a large sphere and for the case of a large oblate (horizontal disc-like) spheroid for multiple trials of each experiment. The small sphere in both cases has a radius of $0.05$ cm. These observations indicate that an attractive force is created by the large body upon the small body when their geometric centers are at the same heights. Observe that the slopes for these curves indicate that the attraction is stronger for the wide spheroid than that of the sphere. This supports the fact that larger discs induce larger attractive forces on smaller particles and gives insight into the collective dynamics of the many particle system: as clusters grow into large-scale discs, their attractive force upon individual particles (or smaller clusters) becomes effectively stronger and stronger until all particles are packed into the single large disc-like shape observed in Fig. 1. In particular, in our experiments presented in Fig. 1, we measure that the attraction speed for a single particle approaching a large cluster can be as large as $20$ times that of the attraction speed between two isolated spheres under similar conditions. See Supplementary Movie 2 for video documenting the collapse for these two cases involving the large sphere/spheroid.

### Mathematical modeling

The theoretical explanation for this unexplored effective force of attraction lies within diffusion-induced flows, first studied by O. M. Phillips^10^ and C. Wunsch^11^. These are flows that originate from a mismatch between the background horizontal isolines of density, which initially intersect the surface non-orthogonally. The no-flux boundary condition for salt transport implies that this angle of intersection must be 90 degrees. As a result, on an initial transient timescale the isolines are bent to locally align with the surface normal. But this produces a lifting (or depression) of density which creates a buoyancy imbalance which, in turn, creates a fluid flow. Of course, the full, quantitative explanation of this behavior relies upon the integration of the partial differential equations (PDEs) underlying the fluid system. Although our studies involve fully three-dimensional flows and non-planar geometry, some intuition can be found for the special case of an infinite tilted flat plane inserted into a linearly stratified fluid: for this geometry there is an exact steady solution^10,11^ to the Navier-Stokes equations coupled to an advection-diffusion equation for salt concentration. This solution shows a boundary layer region in which there is a density anomaly and a shear flow up the top side of the sloped wall. We remark that experimental work of Allshouse and Peacock, using a freely suspended wedge-shaped object, demonstrated that such flows are in fact sufficient to self transport a single object^12,13^. For our studies, the sphere and spheroids are symmetric, and thus no self-induced motion is generated by a single body in isolation. And yet, self-induced flows are generated by these bodies, and those flows induce the collective motion of other nearby bodies, such as documented in Fig. 1. To study theoretically and numerically these flows in detail, we will consider first the case of single body held fixed in background linear stratification, whose diffusion-induced flow can be used as an experimental benchmark; we then move on to the two-body case, which allows us to isolate the attractive force.

The characteristic velocity and length scales for the Phillip’s solution are $U=\kappa {\left(\frac{g\sigma }{\kappa \mu }\right)}^{1/4}$, and $L={\left(\frac{\kappa \mu }{g\sigma }\right)}^{1/4}$, where $g$ is that gravitational acceleration, $\mu$ and $\kappa$ are the dynamic fluid viscosity and salt diffusivity, and $\sigma$ is the slope of the background density field (all assumed to be constant in this work). The length scale $L$ defines the thickness of the boundary layer above the tilted flat plane and the velocity scale $U$ defines its strength. We use this velocity scale to nondimensionalize the equations of motion for the velocity field ${\boldsymbol{u}}$, pressure $P$, density $\rho$ (varying only through the evolution of the salt concentration), position ${\boldsymbol{x}}$, and time $t$, (for a single sphere of radius $a$ for brevity in exposition) via $\tilde{{\boldsymbol{x}}}=\frac{{\boldsymbol{x}}}{a},\tilde{{\boldsymbol{u}}}=\frac{{\boldsymbol{u}}}{U},\tilde{\rho }=\frac{\rho }{\sigma a},\tilde{P}=\frac{Pa}{\mu U}$, and $\tilde{t}=\frac{t\kappa }{{a}^{2}}$. The resulting non-dimensional PDE system (dropping tildes and primes) for the incompressible fluid velocity and concentration field is, respectively:

1 $$Re\,\rho \left[\frac{1}{Pe}\frac{\partial {\boldsymbol{u}}}{\partial t}+{\boldsymbol{u}}\cdot \nabla {\boldsymbol{u}}\right]=-\nabla P-P{e}^{3}\rho \hat{z}+\Delta {\boldsymbol{u}}$$

2 $$\frac{\partial \rho }{\partial t}+Pe\,{\boldsymbol{u}}\cdot \nabla \rho =\Delta \rho$$

where the Reynolds number is $Re=\frac{\sigma {a}^{2}U}{\mu }$ and the Peclet number is $Pe=\frac{a}{L}$. For all the experiments we have run, $Re\;<\; 0.001$, and, as such, the so-called Stokes approximation may be employed, which sets the left hand side of Eq. (1) to zero while retaining nonlinear effects through non-zero $Pe$ in the advection-diffusion of the salt concentration. As such, the only remaining parameter in the system is the Peclet number (of course, when the object is non-spherically symmetric, then its aspect ratio provides an additional non-dimensional parameter). The boundary conditions are assumed to be no-slip for the velocity and no-flux for the tracer on any physical boundary (see Supplementary Note 6 and Supplementary Fig. 7 for exact solutions derived with these as well as other boundary conditions).

Unless otherwise stated, we seek steady solutions of these equations using three different approaches. The steady approximation is well supported through standard partial differential equation estimates, which suggest that the transients will decay after the first hour of our experiment with the large sphere/spheroids, and even faster (on the order of a minute) with the small spheres for the aggregation experiments (total experimental time typically lasting $16$ hours). First, for very small Peclet numbers, this evolution of the salt concentration decouples from the fluid velocity and in fact is, surprisingly, equivalent to finding the irrotational velocity potential for the velocity field induced by a steady vertical flow past a fixed sphere or spheroid. In turn, the actual velocity field is constructed through convolution of the concentration field, $\rho$, with the available Green’s function associated with a point source of momentum in the presence of a no-slip sphere. See Supplementary Note 5 and Supplementary Figs. 5 and 6 for details regarding these solutions, the Green’s function, and the imaging methods employed to generate physical solutions. In this low Peclet limit in free space, we have also derived mathematically exact solutions which satisfy the boundary conditions on a single sphere. These solutions are presented in Supplementary Note 6 and document the possibility of complete flow reversal as the boundary conditions are switched from no-flux (for salt diffusion), to continuity of temperature and heat flux for particles with different heat conductivities from the surrounding fluid.

Next, for general Peclet numbers, we utilize the finite element package COMSOL to calculate steady solutions of these equations of motion. See Supplementary Notes 1 and 2, Supplementary Table 1, and Supplementary Figs. 1 and 2 for extended details on this calculation.

**Fig. 3:**
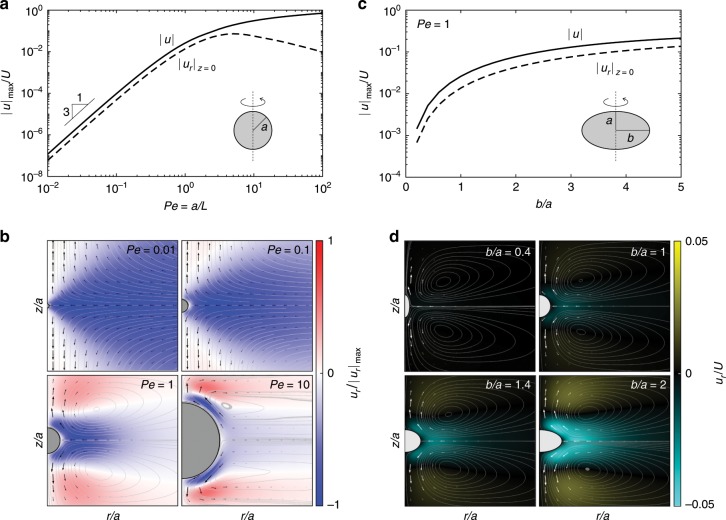
Flow strengths and structures for spheres and spheroids. **a** Overall max velocity and equatorial maximum cylindrical radial speed, ${u}_{r}$ as a function of the Peclet number for spheres. **b** Flow structures induced by a sphere for four different Peclet numbers in a vertical plane slicing the sphere through north and south pole, color representing horizontal velocity scaled by its maximum value for each Peclet number. **c** Overall max velocity and equatorial maximum cylindrical radial speed as a function of the aspect ratio for spheroids. **d** Flow structures induced by four different spheroids, scaled by the Phillips velocity, $U$ at $Pe=1$.

Figure 3 depicts the simulations for this method, documenting the toroidal flow structures observed for a range of Peclet numbers. In the top left panel, we document, for the case of a sphere, the maximum flow speed in the equatorial plane as well as the maximum speed overall in the entire domain as a function of the Peclet number. In the equatorial plane, there is an optimal velocity which occurs at a Peclet number slightly bigger than unity. As a benchmark, observe that the slope (here in log-log coordinates) at low Peclet number is $3$ (i.e. ${\bf{u}} \sim P{e}^{3}$), which is the exact theoretical scaling in this limit. In the lower left panels, the companion flow structures are observed for four representative Peclet numbers. The background color scheme is assigned by the radial velocity, with negative values corresponding to horizontal velocities directed left. Observe that in all cases, in the equatorial plane, the velocities are directed to attract nearby fluid particles towards the sphere. In the right panels of Fig. 3 we show the analogous plots for spheroids of different aspect ratios. In the top, we show the max speed in the equatorial plane and global max speed as a function of aspect ratio, whereas in the bottom, we document the toroidal flow structures for four representative aspect ratios. In all cases, we observe a monotonic increase in the max equatorial speed as the horizontal disc radius increases.

### Comparison of PIV with simulations

**Fig. 4:**
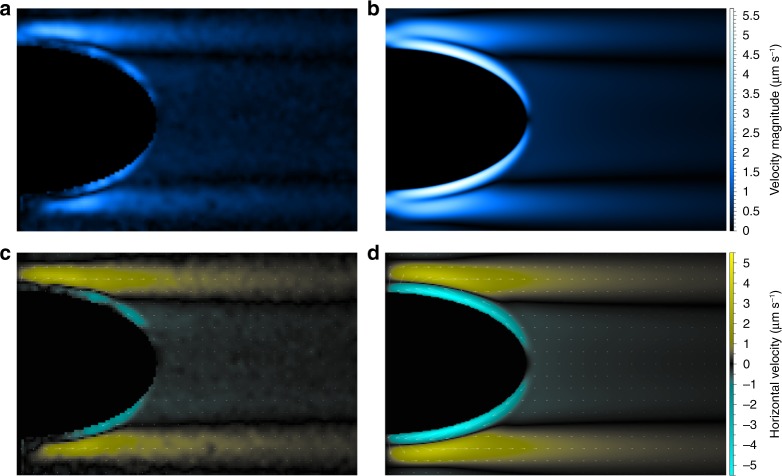
Comparison of experiment with simulation. The flow field in a vertical plane slicing the spheroid through north and south pole with $Pe=52$, $\mu =0.016$ Poise, $\kappa =1.5\,\times\, 1{0}^{-5}$ cm^2^ s^−1^ m, spheroid vertical radius, $0.5$ cm, width, $2$ cm, and with density gradient $\sigma =0.002$ g c^−4^. **a** Flow speed from PIV. **b** Flow speed from COMSOL. **c** Horizontal flow from PIV. **d** Horizontal flow from COMSOL.

In Fig. 4, we present the direct comparison of the flow field from the experiment, measured using PIV, with the COMSOL simulated flow for the parameters of the experiment for the case of our oblate spheroid, see caption for experimental parameters. The strong quantitative agreement between the flow structures and magnitudes clearly supports that the attraction mechanism is indeed hydrodynamic. Please see Supplementary Movie 4 for a video showing the particle evolution in the experiment used to construct the PIV image. By assuming the smaller sphere moves as a passive tracer under the flow induced by the large sphere (or spheroid), we compute the evolution of the separation distance between the large and small bodies. By “passive” we are specifically referring to the assumption that the particle is advected with the local fluid velocity and that its presence does not significantly disrupt the structure of the flow field created by the large particle. This assumption is valid in our case as the small sphere is significantly smaller than the larger particle.

We next apply this method to the experimental observations reported in Fig. 2. For this case involving NaCl in water, $\kappa \simeq 1.5\times 1{0}^{-5}$ cm^2^ s^−1^, the radius $a=0.5$ cm, and $Pe\simeq 41$. These predictions are shown superimposed over the experimental data in the right panel of Fig. 2, where we also show in the left panel the corresponding computed density isolines for the sphere/spheroid. The agreement is quantitative until the objects get sufficiently close together, at which point the passive tracer assumption begins to fail as particle–particle interactions develop.

### Multi-particle interactions and Stokesian dynamics

**Fig. 5:**
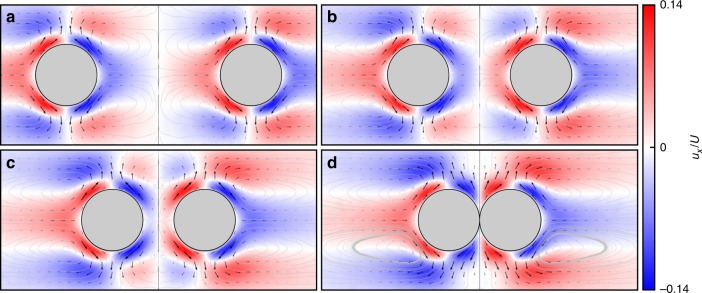
Flow structures created by two spheres. Four different fixed separation distances (**a** $4$, **b** $2$, **c** $1$, and **d** $0$ radii), with flows computed assuming $Pe=5.35$, $\sigma =0.03$ g cm^−4^, and $a=0.045$ cm, scale bar on the right normalized by Phillips velocity $U$.

To explore these particle–particle interactions, we further perform time dependent simulations in COMSOL with two spheres at an array of fixed separation differences (see Supplementary Note 3, Supplementary Table 2, and Supplementary Figs. 2 and 3 for details regarding these simulations) to document the rapid convergence to the steady state behavior alluded to above. This yields both the diffusion-induced flows exterior to two spheres as well as the time evolution of the force exerted on each sphere by these flows, computed through the explicit evaluation of the surface integral of the stress tensor. We note that recent studies have explored anomalous force calculations for particles moving vertically through stratification^18,19^, whereas here we are focusing on horizontal motions. Supplementary Fig. 3 documents how this force rapidly achieves its steady state, further justifying the steady state assumption. Figure 5 displays the flows induced by the two fixed spheres at for four different separation distances after steady state has been reached (approximately one minute for these separation distances) for parameters $Pe=5.1$, $\sigma =0.03$ g cm^−4^, $\mu =0.0113$ Poise, and radii $a=0.045$ cm. The trend is that, for well separated spheres, interactive effects are minimal, as the flow structures resemble the case for an individual sphere from Fig. 3. As the spheres are moved closer together, the sphere-sphere interaction modifies the flow structures, with strong flows between the spheres. These flows create an asymmetric distribution of stress on each sphere, which gives rise to the increased attractive force between the spheres as they approach each other, documented in Supplementary Fig. 2.

**Fig. 6:**
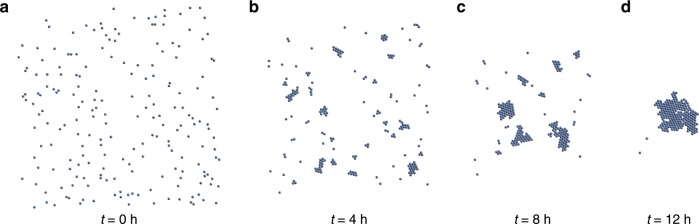
**Modified Stokesian dynamics simulation**. Snapshots of many-body self-assembly and cluster formation at different times (**a** 0 hrs, **b** 4 hrs, **c** 8 hrs, **d** 12 hrs), with $a=0.045$ cm, $\sigma =0.03$ g cm^−4^, $\mu =0.0113$ Poise, $\kappa =1.5\,\times\, 1{0}^{-5}$ cm^2^s^−1^.

With this pairwise force constructed, we may modify standard, available Stokesian dynamics solvers used in the sedimentation community^20,21^ to build many-bodied cluster dynamics simulations. See the Supplementary Note 4 and Supplementary Fig. 4 for details on this implementation. As a benchmark, we experimentally monitor the separation distance between two $0.045$ cm radius spheres in two different linear stratifications, one with $\sigma =0.025$ g c^−4^ and one with $\sigma =0.03$ g cm^−4^, with $\mu =0.0113$ Poise and $\kappa =1.5\times 1{0}^{-5}$ cm^2^ s^−1^. Shown in Supplementary Fig. 4 is the comparison of the experiment with two modified Stokesian dynamics simulations that incorporate the numerically measured two-body diffusion-induced force, which documents quantitative agreement. Next, we apply the modified Stokesian dynamics simulation to a large number of particles. Fig. 6 depicts the evolution at four output times for $200$ identical particles with radius $a=0.045$ cm and force derived from a COMSOL simulation with parameters $\sigma =0.03$ g cm^−4^, $\mu =0.0113$ Poise, and $\kappa =1.5\times 1{0}^{-5}$ cm^2^ s^−1^; this system exhibits self-assembly and cluster formation with many features matching the experiment. The simulation of self-assembly and cluster formation is documented in Supplementary Movie 5.

## Discussion

Of course, further work is needed to account for the full, three-dimensional nature of the diffusion-induced flow and the force that it generates within the Stokesian dynamics approach, as well accounting for subtle effects such as the distribution of sizes and shapes, which are to be expected under natural conditions. Furthermore, future work will aim to explore the aggregation process in more detail: specifically how the size and orientation of an existing aggregates influences the attraction rate of nearby isolated particles.

As mentioned above, we have developed a number of mathematically exact solutions in the zero Peclet limit, which satisfy a menu of different boundary conditions. These solutions provide an alternative starting point to building complete many particle simulations. It is interesting to consider extending the asymptotics of small Peclet regimes for these exact solutions. If a spectral method with physically relevant boundary conditions could be implemented, work by List (and more recently Ardekani and Stocker) could be a starting point for such an extension^16,17^.

In this letter, we have demonstrated an unexplored interaction force, which exists between bodies suspended in a stratified fluid. The force origin lies in a body’s self creation of a diffusion-induced, toroidal flow that attracts nearby matter suspended at the same depth. Our analysis has shown that these flows are quantitatively predicted by solving the Stokes equations coupled to an advection-diffusion equation. We remark that we have also experimentally observed that these flows may induce an effective repulsive force for particles suspended above (or below) a large body, as they are on the opposite (repulsive) side of the toroid (see Supplementary Movie 3). Interestingly, Allshouse and Peacock briefly reported in their supplementary material to have observed that two skinny wedges repel each other without discussion of the flow structures involved. It is likely that that repulsion was similarly a result of the three-dimensionality of the fluid flows generated. We also stress that the phenomena we identified occur over a wide range of different particle materials (glass, photopolymer resin, polystyrene), and we have even observed the same behavior in a non-electrolytic fluid (watery corn syrup). In contrast to self-assembly mechanisms that rely on adjustable parameters, our first-principle theory accurately predicts the source of an attractive force and it allows the extension of Stokesian dynamics to reproduce the experimental clustering observations. We remark that our mechanism is distinct from other small scale effects (electro/diffusiophoresis) in that the particle motion is orthogonal to the background gradient (with velocities scaling sublinearly in $\sigma$ as opposed to linearly^15^). Further, for the case of symmetric bodies, motion is only induced through collective interactions. We also emphasize that this process may be relevant in oceans and lakes, particularly at scales smaller than the Kolmogorov scale of turbulence. Below this scale, which can vary from centimeters (at depth) to tens of microns (in surface water)^8,14^, turbulence is negligible, and these processes may participate in the formation and clustering of marine aggregates. Of course, more work is necessary to address the relevance of these effects in such field applications.

## Methods

### Experimental setup and materials

To create a linear stratification, deionized water is mixed with NaCl, degassed in a vacuum chamber at −24inHg for 10 minutes, and thermalized in a water bath. A two bucket method is used, where fresh water is pumped via Cole-Parmer Ismatec ISM405A pumps at a constant rate into a mixing bucket initially full of saline. Concurrently, this mixture is pumped through a floating diffuser to a depth of ~$10$ cm inside a tank $30.5$ cm tall, $16.5$ cm wide, and $15.5$ cm deep submerged in a custom built Fluke thermal bath to regulate temperature. The short depth walls are made of $1/4$-inch-thick copper to facilitate thermal coupling, and the longer width walls are made of $7/16$ inch acrylic to allow for visualization. To measure the density profile, a Thermo Scientific Orion Star A215 conductivity meter equipped with an Orion 013005MD conductivity cell is carefully submerged into the tank along the wall using a linear stage that measures with $0.01$ mm precision.

Density-matched objects (spheres and/or spheroids of ~$1.05$ g cc^−1^ with radii varying from $50$ microns to centimeter scale) are lowered into the fluid slowly using a thin wire support, with care taken to remove any air bubbles on the surface of the body. Once they have been placed at their buoyant height near the center of the tank, the stratified tank is left for a minimum of $12$ hours to allow decay of transients, which arise from the pouring of the stratification, insertion of the spheres, etc., so that the ambient fluid is quiescent other than for the diffusion-induced flows, which persist for as long as stratification exists (weeks to months). This wait time is sufficient to allow viscous effects to cause any initial fluid motion to come to rest, allowing us to isolate and study the diffusion-induced flows, which are significantly longer lived. From above, images are acquired at $15$, $30$, or $60$ seconds intervals using a Nikon D3 camera equipped with an AFS Micro Nikkor 105 mm lens and a Nikon MC-36A intervalometer. The tank is covered on top with a $1/4$ inch acrylic sheet to reduce evaporation and prevent convective motion.

To manufacture spheres and spheroids, we used a Formlabs Form 2 3D printer with Formlabs Clear V4 photopolymer resin printed at $25$ micron layer thickness. The bodies were printed in halves split along the equatorial plane and joined together with epoxy. An interior cavity in the top half allows for the printed bodies to be density matched to polystyrene tracer spheres of density $\simeq\! 1.05$ g cc^−1^. The polystyrene spheres used for the self-assembly experiments varied in radius from $0.025$ to $0.05$ cm, and the vertical density profile was two-layer (rather than linear as used in the control experiments). Moreover, we note that two sphere experiments were performed with glass spheres of radius $0.25$ cm and similar attraction was observed. We also note that similar attractions were observed in experiments performed in a non-electrolytic corn syrup solution with density stratification achieved by varying water content.

### PIV measurements

To perform particle imaging velocimetry (PIV), a linear stratification is prepared, as described above, with all experimental parameters reported in Fig. 4. We remark that linear stratification provide excellent profiles for benchmarking in that they remain steady except in the vicinity of the top and bottom.The 3D printed spheroid is attached to the tank bottom using a monofilament. Glass microspheres are mixed with salt water matching the top density of the tank and degassed before being released into the tank through a diffuser. In order to reduce transients, the system is left to relax for 24 hours after which PIV is performed with a laser sheet whose normal is oriented horizontally, thus axially slicing the spheroid when entered from the tank side. Imaging is performed with the optical setup described above on $30$ seconds intervals. Two-dimensional, two-component PIV analysis is performed in LaVision DaVis 7.2 software and time averaged due to sparse seeding. A vertical drift owing to settling of seeding particles is measured far from the spheroid and subtracted from the final velocity field. We note that the PIV seems to mildly underestimate the flow strengths as compared with manually tracking individual particles, which could be attributed to the difficulty in seeding the thin boundary layer regions where the flow is strongest.

## Supplementary information

Supplementary Information

Description of Additional Supplementary Files

Supplementary Movie 1

Supplementary Movie 2

Supplementary Movie 3

Supplementary Movie 4

Supplementary Movie 5

## Data Availability

All relevant data are available upon request from the authors.

## Code availability

All relevant code is available upon request from the authors.
