# Supplementary material for: A first-principle mechanism for particulate aggregation and self-assembly in stratified fluids
Source: Nat Commun. 2019 Dec 20;10:5804. doi: 10.1038/s41467-019-13643-y (PMC6925262; doi:10.1038/s41467-019-13643-y)
Supplement: Supplementary file 1 — Supplementary Information [file 41467_2019_13643_MOESM1_ESM.pdf]

Supplementary Information

A first-principle mechanism for particulate aggregation and self-assembly in stratified fluids

Camassa et al.

Supplementary Note 1

Equations of motion

Here we provide details regarding how we employ COMSOL to solve the incompressible Stokes equations coupled to a steady advection-diffusion equation for concentration. Making the steady assumption, we solve the following equations using the COMSOL finite element package, currently in an axisymmetric domain of height $2H$ and radius R:

$$
\begin{aligned}
0 &= -\nabla P - \rho g \hat{z} + \mu \Delta \boldsymbol{u} && (1)\\
\nabla \cdot \boldsymbol{u} &= 0 && (2)\\
\boldsymbol{u} \cdot \nabla \rho &= \kappa \Delta \rho && (3)
\end{aligned}
$$

With boundary conditions on velocity field:

$$
\begin{aligned}
\boldsymbol{u}|_{r=a} &= 0 && (4)\\
u_r|_{r=R} &= 0 && (5)\\
u_z|_{z=\pm H} &= 0 && (6)
\end{aligned}
$$

and boundary conditions on density field:

$$
\begin{aligned}
\nabla \rho \cdot \hat{n}|_{r=a} &= 0 && (7)\\
\nabla \rho \cdot \hat{r}|_{r=R} &= 0 && (8)\\
\rho|_{z=\pm H} &= \rho_o \mp \sigma H && (9)
\end{aligned}
$$

As described in the main text, these are the resulting equations after assuming that the Reynolds number is sufficiently small and that sufficient time has passed so that the transient density dynamics have died off. See the main text for definition of the Reynolds number and for the non-dimensional form of the equations of motion. The timescale for transients can be estimated by the first eigenvalue of the Laplacian with vanishing Neumann boundary conditions exterior to a sphere. For our experiments with spheres of size 0.05cm and diffusivity $\kappa = 1.5 \times 10^{-5}$ cm^2/s, this is approximately one hour, while our experiments last 16 hours. See the evolution of force calculation below in Supplementary Figure 3 for further discussion.

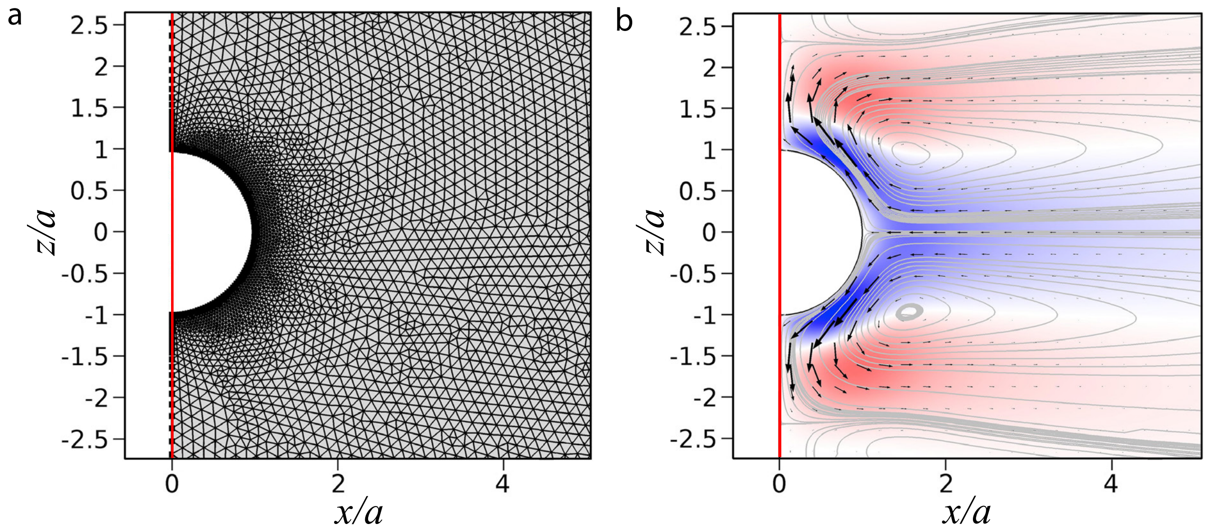

Supplementary Figure 1: COMSOL mesh and output. a: Zoom-in of mesh used for $Pe = 1 - 10$ simulations. b: Predicted flow for $Pe = 5$. The red line indicates the axis of symmetry.

Pe	$R = 2H$	**BL element size**	**Largest element size**	**Element growth rate**
0.01–0.1	750a	0.032a	2.56a	1.025
0.1–1	100a	0.032a	0.64a	1.05
1–10	20a	0.032a	0.16a	1.1
10–31.6	10a	0.008a	0.08a	1.2
31.6–100	4a	0.004a	0.02a	1.4

Supplementary Table 1: Mesh parameters for COMSOL simulations for single sphere.

Supplementary Note 2

Domain and meshing

We chose H and R large enough and mesh element size small enough for each simulation such that the peak speeds (both global maximum speed and maximum horizontal velocity in the equatorial plane) have converged to 3 significant digits. There is a refined region near the surface of the sphere (in the boundary layer) that transitions smoothly with a specified element growth rate to the far-field, where the largest element size is constrained (see Supplementary Figure 1 above). See the main article for definition of the Peclet number. We employ a triangular mesh with these restrictions, see Supplementary Table 1.

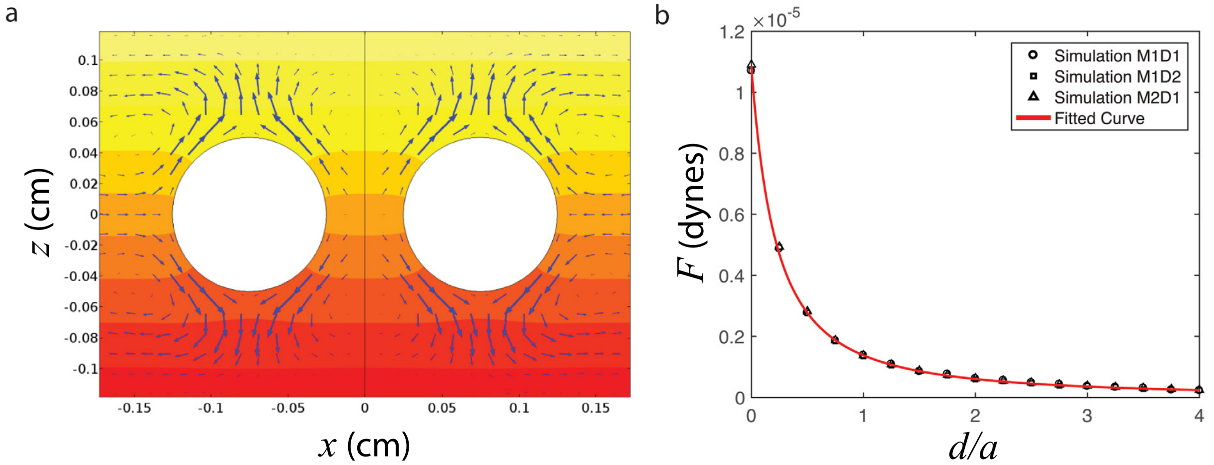

Supplementary Figure 2: COMSOL force calculation. 3D simulations of two identical spheres of radius a spaced by an amount d in linear stratification at $Pe = 5.35$. a: Steady-state density and flow field for two spheres at $d = 0$ (Simulation M2D1) in vertical plane slicing north and south poles. b: Steady attractive force on one sphere computed by numerically integrating the projected stress tensor. Physical parameters used are comparable to the experiment shown in Supplementary Movie 1: $a = 0.045$ cm, $\kappa = 1.5 \times 10^{-5}$ cm^2/s, $\sigma = 0.03$ g/cm^4, $g = 981$ cm/s^2, and $\mu = 0.0113$ Poise.

**Trial**	X	Y	Z	**BL element size**	**Largest size**	**Growth rate**
M1D1	6a+d	4a	8a	0.125a	0.25a	1.1
M1D2	8a+d	6a	12a	0.125a	0.25a	1.1
M2D1	6a+d	4a+2d	8a	0.0625a	0.125a	1.1

Supplementary Table 2: Mesh parameters for COMSOL simulations for force calculation.

Supplementary Note 3

3D unsteady simulations

For the 3D simulations, a solution of the coupled Stokes flow and *unsteady* advection-diffusion equations for density is sought by running to sufficiently long time for transients to decay. Specifically, we numerically solve the coupled equations

$$0 = -\nabla P - \rho g \hat{z} + \mu \Delta \boldsymbol{u}, \tag{10}$$

$$\nabla \cdot \boldsymbol{u} = 0, \tag{11}$$

$$\frac{\partial \rho}{\partial t} + \boldsymbol{u} \cdot \nabla \rho = \kappa \Delta \rho. \tag{12}$$

For the case shown in Supplementary Figure 2, a rectangular domain size of $X \times Y \times Z$ is used, with mirror symmetry conditions about the $y - z$ and $z - x$ planes. Parameters of the domain and mesh are provided in Supplementary Table 2., and the attractive force shows good convergence for both the domain size and mesh resolution used, as evidenced in Supplementary Figure 2(b). The forces are computed by numerically integrating the projected stress tensor which includes both viscous and pressure contributions. We monitor the evolution of this force in time to assess the timescale for decay of transients as a function of the separation distance. This is documented in Supplementary Figure 3 which shows the evolution of the force normalized by its long time limit for 4 different spacings. For particles which are close, the transient decays extremely rapidly, on the order of one minute. Clearly, the diffusion induced flow from one particle will take longer to affect the other particle if their separation distance is large, but the velocities induced when the particles are well separated is very weak, so it may well be the case that the steady assumption is still valid even at large separations. Even for separation distances of 10 diameters, the transient decays in less than two minutes for experiments which take 8 hours to collapse.

The force data shown has a reasonable fit to an expression of the form

$$\frac{F}{F_0} = \left(\frac{d}{a} + 0.3249 \right)^{-1.466} \tag{13}$$

where $F_0 = 2.0865 \times 10^{-6}$ dynes, which is then used for our modified Stokesian dynamics simulations.

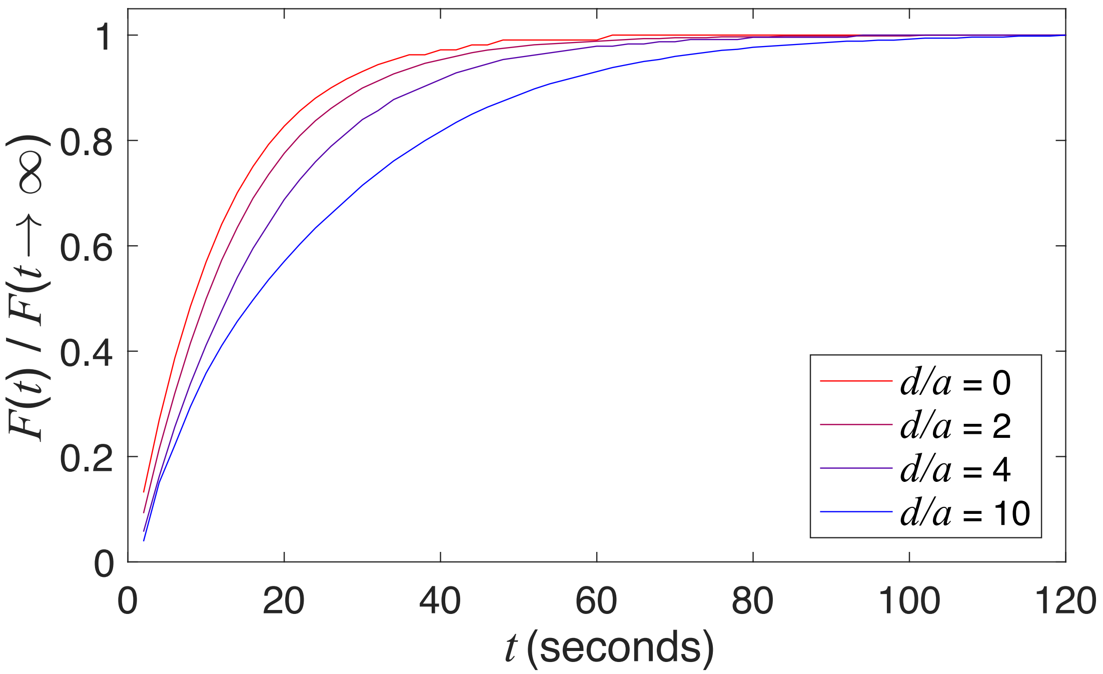

Supplementary Figure 3: Unsteady COMSOL force evolution. Evolution of stress force on one sphere from the diffusion induced flow from two identical spheres of radius $a = 0.045$cm at four different spacings in linear stratification with $\kappa = 1.5 \times 10^{-5}$ cm^2/s, $\sigma = 0.03$ g/cm^4, $\mu = 0.0113$ g/(cm·s), and $g = 981$ cm/s^2.

Supplementary Note 4

Modified Stokesian dynamics

We have modified a Stokesian dynamics software package available on GitHub developed by Townsend (*4*) which implements the formalism pioneered by (*5*). This solver allows for a user defined external force field. In our implementation, we use the above force field expression (13) with a correction that accounts for the relative velocity of the moving particle with respect to the diffusion induced flow. This correction is a non-trivial modification of the algorithm, as it requires introducing semi-implicit time-stepping in the computation of velocities to avoid numerical instabilities. The algorithm is applied to a set of two small experiments as a benchmark. Supplementary Figure 4 depicts the separation distance as a function of time constructed with modified Stokesian dynamics compared to two experiments with spheres of radius 0.045cm in two different linear background stratifications ($\sigma = 0.03$, and $\sigma = 0.025\text{g/cm}^4$), with viscosity and diffusivity as in Supplementary Figure 3. With the strong agreement observed in this benchmark, the algorithm may be applied to hundreds of spheres, as we have demonstrated in the main body. We note that improvements can be made by running the two sphere calculation in a moving frame to represent more faithfully the coupled nature of the advection-diffusion dynamics. In future work we plan to report on these more refined approaches in detail.

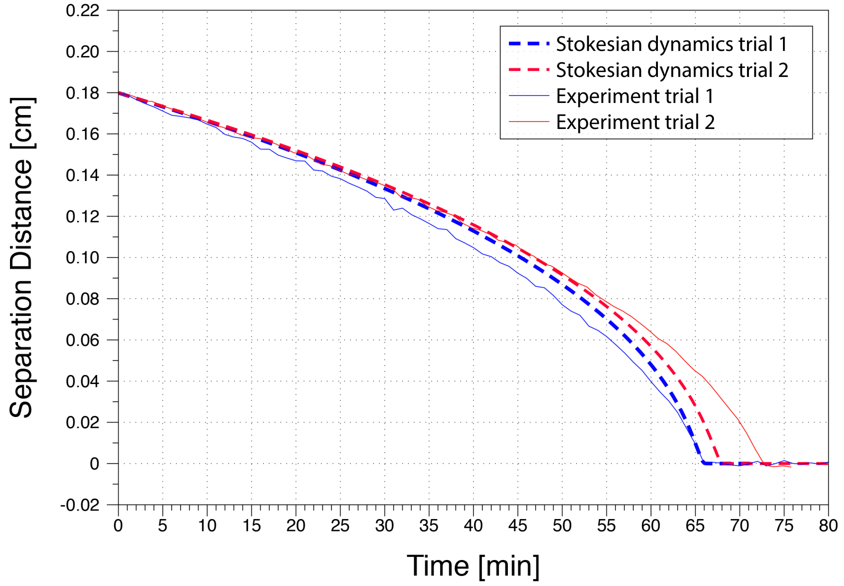

Supplementary Figure 4: Stokesian dynamics (SD) compared with two-sphere experiments. SD modified by adding the two-body force computed via COMSOL given in force field expression (13). Here, $a = 0.045$ cm, with two linear background density fields, $\sigma = 0.03\text{g/cm}^4$ and $\sigma = 0.025\text{g/cm}^4$ in the first and second trial respectively, other experimental parameters matching Supplementary Figure 3.

Supplementary Note 5

Low Peclet number limit

We next discuss a semi-analytical approach (valid at low Peclet number) which exactly computes the density perturbation from the background for a single body and approximately for multiple bodies using the method of images. The flow is then computed using convolution with a special Green's function augmented again by the method of images.

In the low Peclet number limit for the non-dimensionalization presented in the main text, the advection diffusion equation for the concentration field, ρ, reduces to a simple heat equation, which once transients decay (see prior timescale discussion), further reduces to Laplace's equation, with flow given by solution to the Stokes equations:

$$\Delta\rho = 0 \tag{14}$$
$$0 = -\nabla P - Pe^3 \rho g \hat{z} + \mu \Delta \boldsymbol{u} \tag{15}$$
$$\nabla \cdot \boldsymbol{u} = 0 \tag{16}$$
$$\tag{17}$$

with boundary conditions being impermeable for the concentration, and no-slip for the velocity.

For the case of the density perturbation itself, with a linear background density profile, explicit formulae are available: Interestingly, in this limit, the concentration field problem is actually equivalent to computing the induced potential field for a body held in a vertical constant flow, under the assumption of potential flow. For a single sphere (*1*):

$$\rho = \sigma \frac{a_1^3 z}{2\left(x^2+y^2+z^2\right)^{3/2}} \tag{18}$$

where a_1 is the radius of the sphere. In the presence of another sphere of radius a_2, a correction to the density anomaly is given by (*2*)

$$\rho_{corr} = \sigma \frac{a_1^3 a_2^3 z}{4c^3\left(x^2+y^2+z^2\right)^{3/2}} \tag{19}$$

where c is the distance between centers (the companion sphere is obtained through summing identical translated formulae). The density anomaly for the oblate spheroid is given by (streamfunction in (*1*), potential through our own calculation)

$$\rho = \sigma \frac{a^2\sqrt{a^2-b^2}\cos(\eta)\left(\sinh(\xi)\cot^{-1}(\sinh(\xi))-1\right)}{b\sqrt{a^2-b^2}-a^2\cos^{-1}\left(\frac{b}{a}\right)} \tag{20}$$

where a is the semi-major axis, b is the semi-minor axis, and (ξ, η, θ) are the oblate spheroidal coordinates that define the cartesian mapping $x = \sqrt{a^2-b^2}\sin(\eta)\cosh(\xi)\cos(\theta)$, $y = \sqrt{a^2-b^2}\sin(\eta)\cosh(\xi)\sin(\theta)$, and $z = \sqrt{a^2-b^2}\cos(\eta)\sinh(\xi)$.

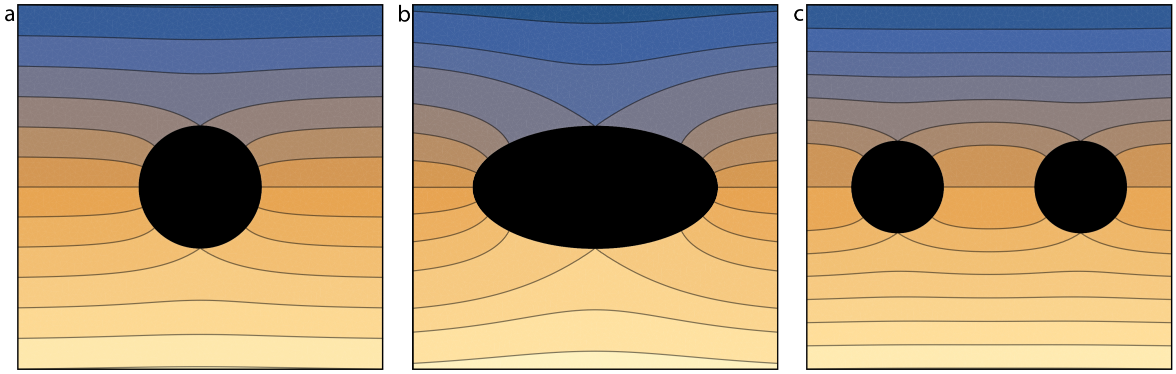

Supplementary Figure 5: Exact density fields in the low Peclet limit. Cases include a: single sphere, b: single spheroid, and c: double sphere (retaining just two reflections).

In Supplementary Figure 5, we present the density anomalies for these cases: on the left is a single sphere, in the middle is an oblate spheroid, and in the right is the case with two spheres. A horizontal wall (lid) may be implemented exactly by adding the solution arising from an imaging sphere positioned at twice the distance between the real sphere's center and the plane. A second horizontal wall (the bottom) may be approximated by repeating this procedure.

Low Peclet number limit

The fluid flow problem is then solved using an exact Green's function derived by Oseen (*3*) for a delta function of momentum inserted into a fluid in the presence of a fixed, no-slip sphere. With this explicit Green's function (given below), we may express the velocity field as a convolution with the exact concentration field:

$$u_i(\boldsymbol{x}) = \int_\Omega G_i(\boldsymbol{x}, \boldsymbol{y})\rho(\boldsymbol{y})g d\boldsymbol{y} \tag{21}$$

where $\boldsymbol{x}$ is the point at which the flow will be evaluated, $\mathbf{y}$ is the integration variable which represents the location of the point force, Ω is all of $\mathbb{R}^3$ exterior to the sphere, and G_i is the Green's function, defined $G_i(\boldsymbol{x}, \boldsymbol{y}) = gT_{i3}/8\pi\mu$, with

$$T_{jk} = \frac{\delta_{jk}}{r} + \frac{(x_j - y_j)(x_k - y_k)}{r^3} - \frac{A}{|\boldsymbol{y}|}\frac{\delta_{jk}}{r^*} - \frac{A^3}{|\boldsymbol{y}|^3}\frac{(x_j - y_j^*)(x_k - y_k^*)}{r^{*3}}$$

$$-\frac{|\boldsymbol{y}|^2 - A^2}{|\mathbf{y}|}\left\{\frac{y_j^* y_k^*}{A^3 r^*} - \frac{A}{|\boldsymbol{y}|^2 r^{*3}}[y_j^*(x_k - y_k^*) + y_k^*(x_j - y_j^*)] + \frac{2y_j^* y_k^*}{A^3}\frac{y_l^*(x_l - y_l^*)}{r^{*3}}\right\}$$

$$-(|\boldsymbol{x}|^2 - A^2)\frac{\partial \varphi_k}{\partial x_j} \tag{22}$$

$$\frac{\partial \varphi_k}{\partial x_j} = \frac{|\boldsymbol{y}|^2 - A^2}{2|\boldsymbol{y}|^3}\left(\frac{-3y_k}{Ar^*} + \frac{A(x_k - y_k^*)}{r^{*3}} + \frac{2y_k}{A}y_j^*\frac{\partial}{\partial y_k^*}\log\frac{|\boldsymbol{y}^*|r^* + x_j y_j^* - |\boldsymbol{y}^*|^2}{|\boldsymbol{x}||\boldsymbol{y}^*| + x_j y_j^*}\right) \tag{23}$$

where

$$\boldsymbol{y}^* = \frac{A^2}{|\boldsymbol{y}|^2}\boldsymbol{y} \tag{24}$$

$$r = |\boldsymbol{y} - \boldsymbol{x}| \tag{25}$$

$$r^* = |\boldsymbol{y}^* - \boldsymbol{x}| \tag{26}$$

We note that the slow decay of the kernel generally will lead to divergences in free space. Imaging the Green's function G_i with respect to two horizontal planes allows for the convolution domain to be limited to an infinitely wide channel of finite height, which is sufficient to restore convergence because of the far field decay rate of the density anomaly.

To represent a vertical no-flux wall at horizontal distance h from the center, which is equivalent to the two-sphere problem with a second sphere located at a position $2h$, we introduce a second image of this type

$$u_i(\boldsymbol{x}) = \int_\Omega (G_i(\boldsymbol{x}, \boldsymbol{y}) + G_i(\boldsymbol{x}_{vert}, \boldsymbol{y}_{vert}))\rho(\boldsymbol{y})g d\boldsymbol{y} \tag{27}$$

where the domain of integration is exterior to the sphere and truncated at the wall, $\boldsymbol{x}_{vert} = (x_1 - 2h, x_2, x_3)$, and $\mathbf{y}_{vert} = (-y_1, y_2, y_3)$. To represent a horizontal no-flux wall at distance h, we introduce a second image as

$$u_i(\boldsymbol{x}) = \int_\Omega (G_i(\boldsymbol{x}, \boldsymbol{y}) - G_i(\boldsymbol{x}_{horiz}, \boldsymbol{y}_{horiz}))\rho(\boldsymbol{y})g d\boldsymbol{y} \tag{28}$$

where the domain of integration is exterior to the sphere and truncated at the wall, $\boldsymbol{x}_{horiz} = (x_1, x_2, x_3 - 2h)$, and $\mathbf{y}_{horiz} = (y_1, y_2, -y_3)$. For our computation, we include an image representing a second sphere shifted in the horizontal direction, and then image both of these above and below to model a top and bottom wall, for a total of six images. The subsequent calculations are performed using a midpoint Riemann sum.

Supplementary Figure 6 displays the flows induced by two fixed spheres in the low Peclet limit for four different separation distances. The trend is that, for well separated spheres, interactive effects are minimal, as the flow structures resemble the case for an individual from main article Figure 3. As the spheres are moved closer together, the sphere-sphere interaction modifies the flow structures, with the flow immediately between the spheres weakening as they approach each other (note the method of images induces some mild errors near the boundaries, but is faithful away from boundaries). Ultimately, as the spheres touch, the flow qualitatively resembles that of a single prolate spheroid and produces the strongest flow of the four spacings.

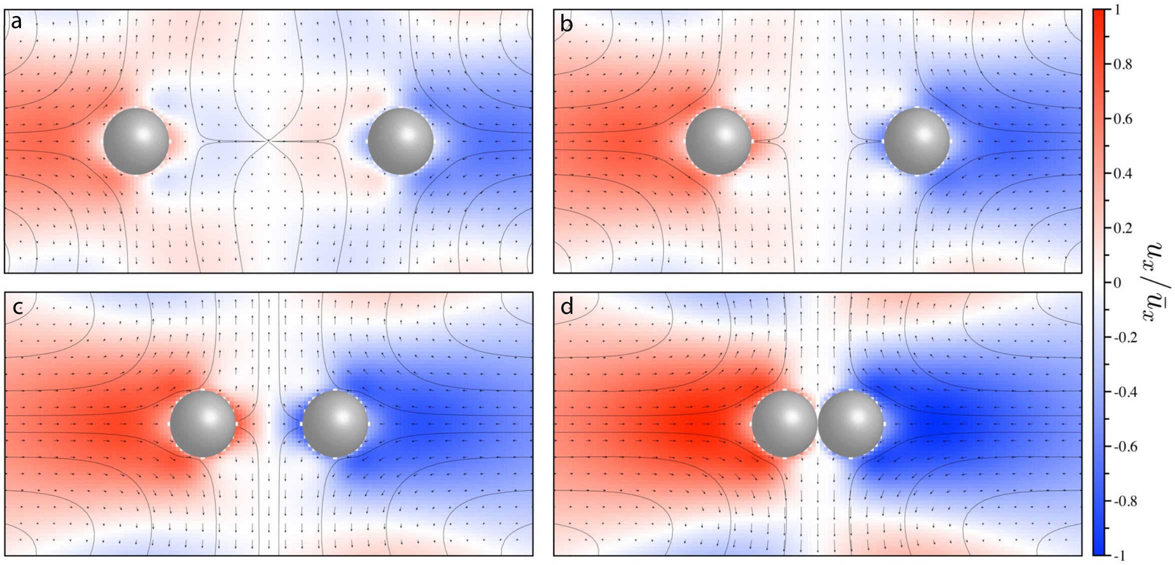

Supplementary Figure 6: Zero Peclet flows from Green's function method for two spheres. Velocity vectors and streamlines created by two spheres at four different fixed separation distances (a: $6a$, b: $4a$, c: $2a$, d: $0a$), with flows computed in the low Peclet number asymptotic limit, scale bar on the right normalized by the maximum of max horizontal speed over the four separation distances.

Supplementary Note 6

Exact solutions in the low Peclet limit

Here we present mathematically exact solutions of the equations of motion in the low Peclet number limit for a single sphere in linear stratification. These include a no-slip boundary condition for the velocity field and a variety of boundary conditions for the stratifying agent. We expect these solutions to be useful building blocks for designing modified Stokesian dynamics simulations at small Peclet numbers. Further, they illustrate how different boundary conditions can reverse the flows and hence lead to different aggregation dynamics.

In spherical coordinates (r, θ, ϕ), the velocity field is represented as

$$\boldsymbol{u} = \nabla \times \left(\frac{\psi(r,\theta)}{r \sin\theta} \hat{\phi} \right) \tag{29}$$

equivalently, $u_r = \frac{\psi_\theta}{r^2 \sin\theta}$, and $u_\theta = -\frac{\psi_r}{r \sin\theta}$

No slip, zero flux boundary conditions

When the bodies are impermeable to the stratifying agent, such as salt, the appropriate boundary conditions on the body are no-flux (vanishing normal derivative). The ensuing exact solutions need to be augmented by the proper matched asymptotics to satisfy physical boundary conditions at infinity, a ubiquitous feature of all low Reynolds number flow mathematics.

Letting $\sigma > 0$, and radius, a, the exact solution is

$$\rho(r,\theta) = -\sigma \left(r\cos\theta + \frac{a^3 \cos\theta}{2r^2} \right) \tag{30}$$

$$\psi(r,\theta) = \frac{\sigma g}{\mu} \left(\frac{a^3 r^2}{32} + \frac{a^7}{32 r^2} - \frac{a^5}{16} \right) \sin\theta \sin 2\theta \tag{31}$$

Shown in Supplementary Figure 7 a, b are the flow and density isolines for a representative case.

No slip, conductive boundary conditions

For bodies that are permeable to the stratifying agent (such as heat with solid-fluid transitions, or salt in the presence of porous media like marine snow), the appropriate boundary condition is continuity of flux and concentration on the body's surface.

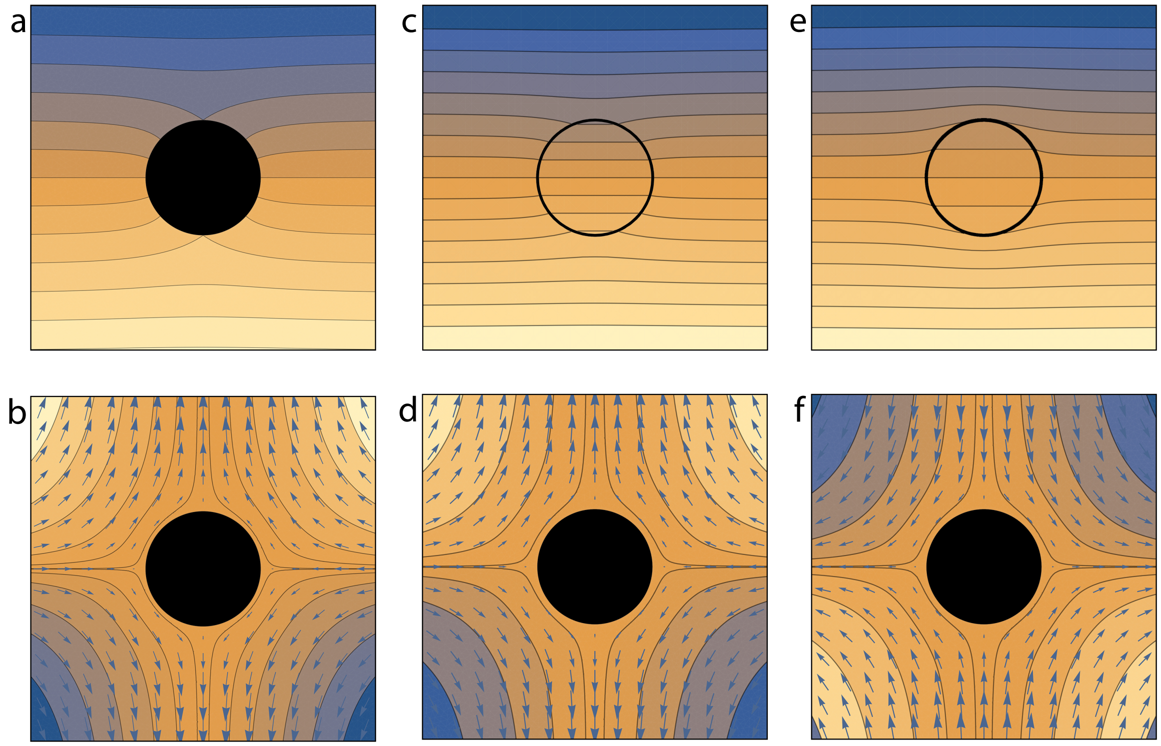

Supplementary Figure 7: Impermeable case: a: density field, b: flow field and streamlines, axial slice. Insulating case, $\kappa_s = 1$, $\kappa_f = 2$: c: density field, d: flow field and streamlines, axial slice. Conducting case, $\kappa_s = 2$, $\kappa_f = 1$: e: density field, f: flow field and streamlines, axial slice.

For a sphere with diffusivity κ_s in a fluid with diffusivity κ_f, the exact solutions now are:

$$\kappa_s \frac{\partial \rho_s}{\partial r}\bigg|_{r=a} = \kappa_f \frac{\partial \rho_f}{\partial r}\bigg|_{r=a} \tag{32}$$

$$\rho_s|_{r=a} = \rho_f|_{r=a} \tag{33}$$

$$\rho_s(r,\theta) = \frac{-3\sigma\kappa_f}{2\kappa_s + \kappa f} r\cos\theta \tag{34}$$

$$\rho_f(r,\theta) = -\sigma\left(r\cos\theta + \frac{a^3(\kappa_f - \kappa_s)}{2\kappa_s + \kappa f}\frac{\cos\theta}{r^2}\right) \tag{35}$$

$$\psi_f(r,\theta) = \frac{\sigma g}{\mu}\left(\frac{a^3(\kappa_f - \kappa_s)}{2\kappa_s + \kappa f}\right)\left(\frac{r^2}{16} + \frac{a^4}{16r^2} - \frac{a^2}{8}\right)\sin\theta\sin 2\theta \tag{36}$$

Shown in Supplementary Figure 7 c, d are the flow and density isolines for a representative case in which the diffusivity of the solid is lower than that of the liquid phase. Conversely, shown in Supplementary Figure 7 e, f are the flow and density isolines for a representative case in which the diffusivity of the solid is higher than that of the liquid phase. Note that the flow has completely reversed in this case, suggesting that this setup could favor aggregation prevalently oriented vertically.

Supplementary References

1. Batchelor, G. K. *An Introduction to Fluid Dynamics*, Cambridge U.P., Cambridge, U.K. (1967)

2. Milne-Thomson, L. M. *Theoretical Hydrodynamics*, Macmillan & Co. Ltd., London, U.K. (1938)

3. Oseen, C. W. *Hydromechanik*, Leipzig, (1927)

4. A. K. Townsend, *The mechanics of suspensions*, Doctoral thesis, University College London, 2017.

5. J. F. Brady and G. Bossis, Stokesian Dynamics, *Ann. Rev. Fluid. Mech.*, **20**, 111-157, 1988.
