# Supplementary material for: A first-principle mechanism for particulate aggregation and self-assembly in stratified fluids
Source: Nat Commun. 2019 Dec 20;10:5804. doi: 10.1038/s41467-019-13643-y (PMC6925262; doi:10.1038/s41467-019-13643-y)
Supplement: Supplementary file 2 — Description of Additional Supplementary Files [file 41467_2019_13643_MOESM2_ESM.pdf]

# Description of Additional Supplementary Files

File Name: Supplementary Movie1
Description: Top view of particles suspended in sharp stratification displaying self-assembly and cluster formation.

File Name: Supplementary Movie 2
Description: Top view of control experiments in linear stratification (as in Figure 2) comparing the rate at which a tracer particle is a attracted to a large sphere versus a large oblate spheroid of the same height.

File Name: Supplementary Movie 3
Description: Demonstration of repulsion of a tracer particle below the equatorial plane from a large oblate spheroid (top and side view).

File Name: Supplementary Movie 4
Description: Tracer particles advected by an oblate spheroid in linear stratification. Illuminated plane intersect axis of rotational symmetry.

File Name: Supplementary Movie 5
Description: Stokesian dynamics simulation of hundreds of spheres using two-body force calculated in COMSOL.
